# Versatile in vitro assay to recognize Cas9‐induced mutations

**DOI:** 10.1002/pld3.269

**Published:** 2020-09-28

**Authors:** Heinrich Bente, Ortrun Mittelsten Scheid, Mattia Donà

**Affiliations:** ^1^ Gregor Mendel Institute of Molecular Plant Biology Austrian Academy of Sciences Vienna BioCenter (VBC) Vienna Austria

**Keywords:** cost and labor‐saving protocol, CRISPR/Cas9, genotyping protocol, in vitro cleavage, mutagenesis, sgRNA cleavage efficiency

## Abstract

The discovery of CRISPR/Cas9 has revolutionized molecular biology, and its impact on plant biotechnology and plant breeding cannot be over‐estimated. In many plant species, its application for mutagenesis is now a routine procedure––if suitable target sites, sufficient expression of the Cas9 protein, and functioning sgRNAs are combined. sgRNAs differ in their efficiency, depending on parameters that are only poorly understood. Several software tools and experience from growing databases are supporting the design of sgRNAs, but some seemingly perfect sgRNAs turn out to be inefficient or fail entirely, and most data bases stem from work with mammalian cells. Different in vitro assays testing sgRNAs in reconstituted Cas9 complexes are available and useful to reduce the risk of failure, especially in plants when CRISPR/Cas9 application requires modifications within the germ line and laborious transformation protocols. Low sgRNA efficiency and long generation times in plants can also contribute to the workload and costs of screening for the wanted genome edits. Here, we present a protocol in which a simple, initial in vitro test for suitable sgRNAs is modified to accelerate genotyping of Cas9‐induced mutations. We demonstrate applicability of our protocol for mutagenesis and mutation screen for specific genes in Arabidopsis, but the principle should be universally suitable to provide a simple, low‐cost, and rapid method to identify edited genes also in other plants and other organisms.

## 1 INTRODUCTION

Mutagenesis is a powerful method to alter an organism's phenotype or to study the function of gene products by disrupting their genomic template. Classical mutagenesis is based on the random distribution of genetic lesions induced by radiation or genotoxic chemicals. While these procedures are valuable approaches for unbiased forward genetic screens or for generating genetic diversity for plant breeding without requiring particular reagents or procedures, they come at the price of creating multiple, unpredictable, and untargeted mutations that need to be screened, separated from unwanted lesions, mapped, and characterized in laborious, time‐ and cost‐intensive procedures. Recently, the precise editing of genomic information within living organisms at single, pre‐defined sites has tremendously enriched the mutagenesis toolbox (Bortesi et al., 2016; Wang, La Russa, & Qi, 2016). While gene replacement remains difficult in plants, working efficiently in only a limited number of experimental systems, targeted disruption of individual genes has become largely routine, opening new avenues for progress in research, breeding, and biotechnology. This advance is based on creating DNA strand breaks by tethering engineered endonucleases to well‐defined target sites. The lesions are then repaired by endogenous proteins, and imprecise repair causes point mutations, deletions, or insertions of variable length and nature, which leads to altered and mostly non‐functional gene products. Although Zinc finger proteins (ZnFs) and transcription activator‐like effectors (TALEs) have been successfully applied to direct endonucleases to specific target sites (Gaj, Gersbach, & Barbas, 2013; Ma, Chen, Blackburn, & Ekker, 2016; Sorlien, Witucki, & Ogas, 2018), the discovery of the CRISPR/Cas9 (clustered regularly interspaced short palindromic repeats) system revolutionized the field (Jinek et al., 2012). Its advantages over the previous approaches lie in its ease of use and adaptability, as the same protein component can be used for many different targets by simply varying the gRNA component that determines the sequence specificity, substantially reducing the effort required to generate the necessary plasmid constructs. The frequency of successful mutagenesis depends on sufficient expression of the Cas protein, a suitable genomic target site with a protospacer adjacent motive (PAM), and a well‐designed single guide RNA (sgRNA). If co‐expressed, the Cas protein and sgRNA assemble via the scaffold sequence of the sgRNA and form a functional ribonucleoprotein complex (RNP) that is targeted to the genomic site by the specific and complementary portion of the sgRNA. There, the Cas protein induces exactly positioned lesions in the DNA strands. These are subsequently repaired by different mechanisms, often resulting in mutations. Numerous articles and reviews document the nearly universal applicability of this system (Chen, Wang, Zhang, Zhang, & Gao, 2019; Saha et al., 2019; Zhang, Deng, Li, Zhang, & Li, 2016). Improved techniques of tissue culture and the versatility of CRISPR/Cas9 systems allow introducing precise mutations in non‐model plants and challenging crop species. Global distribution of CRISPR/Cas9 allows genome editing even with limited financial resources, and its combination with *in planta* meristem induction opens new and faster applications (Maher et al., 2020). Nevertheless, simplifying and optimizing any step in the genome editing procedure can improve the success and reduce labor and costs.

The protein component, especially the most widely applied endonuclease Cas9, is available in several optimized and codon usage‐adapted versions and is the more “reliable” partner in the system, making the sgRNAs the main determinants of success or failure of targeted mutagenesis (Agarwal et al., 2018; Hsu et al., 2013). To optimize sgRNA design, various in silico tools are available which assess the potential efficiency based on numerous features, including position to the next PAM site, GC content, formation of secondary structures, and mismatches. Many software tools also compute the risk that sgRNAs will bring the Cas protein to other genomic positions, thereby creating unwanted off‐target mutations. In silico sgRNA design is an important, though often insufficient step, as queries with the same target region to different software can suggest quite different sgRNAs, and the same sgRNA might receive divergent efficiency scores with different programs (Haeussler et al., 2016). Moreover, most software tools for sgRNA design use algorithms trained with data from animal and human studies, whereas a validated sgRNA‐designing tool for precise genome‐editing in plants is still missing (Naim et al., 2020; Uniyal, Mansotra, Yadav, & Kumar, 2019). Confirmative assessment of sgRNA efficiency, especially if mutagenesis involves lengthy and/or expensive transgenesis, is therefore often obtained by in vitro assembly of candidate sgRNAs with recombinant Cas9 protein and testing the nuclease activity of this RNP on DNA fragments with the target site, resembling a restriction enzyme assay. Many protocols of different complexity are available and applied to exclude non‐efficient combinations (Cong et al., 2013; Dang et al., 2015; Doench et al., 2014; Grainger et al., 2017; Guschin et al., 2010; Liu, Yu, Zhou, & Xing, 2016; Liu, Homma, et al., 2016; Matson, Hosny, Swanson, Hering, & Burlak, 2019; Zhang, Lang, & Zhu, 2018). PCR and CRISPR digestion were combined to genotype mutations in mammalian cell culture (Kim, Kim, Kim, & Kim, 2014) and in transiently transfected protoplast and genome‐edited plants, comparing it with restriction enzyme analysis with amplicons from previously characterized homozygous mutants (Liang, Chen, Yan, Zhang, & Gao, 2018). Based on a simple, fast, versatile, and cheap sgRNA efficiency assay routinely used in our lab, we have adapted the principle to support recognition and validation of successfully mutated sites among pools of genomic DNA. Reconstituted Cas9‐sgRNA complex is incubated with mixed genomic DNA of mutant candidates, cleaving non‐modified sequences, after which subsequent PCR around the target region can provide positive evidence for the presence of mutated sites. This protocol requires solely components/reagents from the initial test of sgRNAs, making it very cost‐efficient. We demonstrate that this (1) is applicable to pools of genomic DNA from more than 10 individuals, (2) operates at different temperatures and in different buffers and with commercially available components, (3) is sensitive to just a single base mutation, (4) can give first hints to the type of mutation obtained, and (5) is fast, versatile, inexpensive, and allows versatile downstream processing of the samples. As a proof of concept, we demonstrate the application for knocking out two specific genes in *Arabidopsis thaliana*, but the principle is transferrable to other target sequences or organisms.

## 2 MATERIAL AND METHODS

### 2.1 Plant growth conditions

Mutagenesis was performed in *Arabidopsis thaliana* plants of the Wt accession Columbia‐0 (Col‐0) or mutants lacking functional ATM (AT3G48190, SAIL_1223_B08) or ATR (AT5G40820, SALK_127346) genes. Seeds were aliquoted in open 2 ml tubes and surface‐sterilized by 10 min exposure to chlorine gas produced by mixing 100 ml of 14% sodium hypochlorite with 10 ml of 37% HCl in a desiccator. Sterilized seeds were stratified at 4°C for two days and plated either on agar‐solidified germination medium (GM) or on soil. Plants were grown at 21°C with 130 µM/m^2^/s light intensity under long day conditions (16 hr light/8 hr dark).

### 2.2 Generation of CRISPR Cas9‐expressing plants

Plants were grown for 4 weeks until they reached the flowering stage. They were then transformed via the floral dip method (Clough & Bent, 1998). We used *Agrobacterium tumefaciens* GV3101 pMP90, harboring a Cas9 expression vector based on the pDE‐Cas9 vector (Fauser, Schiml, & Puchta, 2014) with the following modifications: the Ubiquitin10 promoter was replaced by the egg cell‐specific promoter EC1.2p (Wang et al., 2015) to regulate Cas9 expression and we inserted an additional marker conferring seed fluorescence, based on expression of recombinant OLE1::GFP protein driven by its endogenous promoter (Shimada, Shimada, & Hara‐Nishimura, 2010). sgRNAs were cloned to be expressed under the control of the Arabidopsis U6‐26 snoRNA promoter in a tRNA‐multiplex system as described (Xie, Minkenberg, & Yang, 2015). Briefly, the presence of Arabidopsis tRNA^Gly^ sequences causes editing of the polycistronic transcript into several sgRNAs that are then loaded onto the Cas9 protein. The vector described above (pDEEC::Cas9::OLE1, for short pDEECO) contains a unique *Mlu*I restriction sequence that allows inserting the multiplexed sgRNA cassette with either conventional restriction/ligation or Gibson assembly. A scheme of the construct is shown in Figure S1 and all sgRNAs used in this study are listed in Table S1.

### 2.3 Genomic DNA extraction

Rapid extraction of genomic DNA (gDNA) from two‐week‐old seedlings was performed using a protocol modified after Edwards, Johnstone, and Thompson (1991). In brief, a 5 x 5 mm piece of leaf tissue from a single plant was placed in 500 µl fast‐prep DNA elution buffer (200 mM Tris pH 7.5, 250 mM NaCl, 25 mM EDTA) in a 2 ml tube, ground with 5 glass beads (3.5 mm diameter) for 1 min at 30 Hz in a homogenizer (MM400, Retsch), and subsequently centrifuged for 1 min at 10000 *g*. 400 µl of the supernatant were removed and DNA was precipitated by adding one volume of isopropanol and 1/10 volume of sodium acetate (NaAc; pH 5.2), incubating for 5 min at room temperature, and centrifuging for 5 min at 16000 *g* at 4°C. The DNA pellet was washed once with 70% EtOH. After removal of the supernatant, the pellet was air‐dried and resuspended in 70 µl TE, dissolved thoroughly by pipetting, and stored at −20°C until use.

For the genotyping assay, we generated pools with nine plants each by collecting one leaf of similar size from each plant into the same tube containing 5 glass beads. Samples were frozen in liquid nitrogen and ground to fine powder as described above. DNA was extracted using CTAB and phenol/chloroform extraction as described by Clarke (2009). Briefly, 300 µl CTAB buffer was added to the frozen ground tissue and, after addition of RNase A (EN0531, Thermo Fisher Scientific) to a final concentration of 10 µg/ml, samples were incubated on a shaker for 1 hr at 65°C. DNA was then purified by phenol/chloroform extraction and precipitated with ice‐cold isopropanol according to standard procedures. The DNA pellet was washed once with 70% ethanol, air‐dried, and resuspended in 70 µl TE buffer. DNA concentration was measured using a Nanodrop ND1000 spectrophotometer (Thermo Fisher Scientific).

### 2.4 sgRNA sequence prediction and primer design

Suitable sgRNA sequences were predicted with three in silico tools: CHOPCHOP v2, CCTOP, and CRISPR‐P v2.0 (Labun, Montague, Gagnon, Thyme, & Valen, 2016; Liu et al., 2017; Stemmer, Thumberger, Keyer, Wittbrodt, & Mateo, 2015) to target the genes *RDR2* (AT4G11130), *RDR6* (AT3G49500), *AGO4* (AT2G27040), *NRPD1a* (AT1G63020), *NRPE1* (AT2G40030), *ADH1* (AT1G77120), *CHLI1* (AT4G18480), and *PIE1* (AT3G12810) of *Arabidopsis thaliana*.

### 2.5 sgRNA preparation

Templates for the in vitro transcription of sgRNAs were amplified from vectors containing the initially described sgRNA scaffold sequence (Jinek et al., 2012) with Q5 DNA polymerase (M0491, NEB) according to the suppliers’ instructions, with 61°C annealing temperature and 30 cycles, using an sgRNA‐specific forward primer and the universal scaffold reverse primer (Table S1). Amplicons were confirmed by gel electrophoresis and optionally purified using DNA Clean and Concentrator‐25 (Zymo Research D4033).

sgRNAs were transcribed from the amplified PCR products in vitro with T7 RNA polymerase (P2075, Promega, in the present work provided by the IMP/IMBA/GMI Molecular Biology Core Facility). One microliter of PCR product was used in a total reaction volume of 20 µl containing 1 x T7 transcription buffer (50 mM Tris pH 7.5, 15 mM MgCl2, 5 mM dithiothreitol (DTT), 2 mM spermidine), 2 mM NTPs, and 1 µl of 54 mg/ml T7 RNA polymerase and incubated overnight at 37°C. Afterwards, 1 U of DNase I (EN0525, Thermo Fisher Scientific) was added and incubated for 30 min at 37°C. The DNase reaction was stopped by adding 2 µl 50 mM EDTA and heating to 65°C for 15 min. Two microliters of the reaction were loaded onto 1.5% TAE‐agarose gels to assess the efficiency of transcription. sgRNAs were purified with RNA Clean and Concentrator (D1017, Zymo Research). sgRNA concentration was measured with a Nanodrop ND1000 spectrophotometer (Thermo Fisher Scientific) and adjusted to 30 ng/µl with RNase‐free ddH_2_O. Synthesised sgRNAs were aliquoted, flash‐frozen in liquid nitrogen, and stored at −80°C until use.

### 2.6 Generating templates for in vitro cleavage assays

**FIGURE 1:**
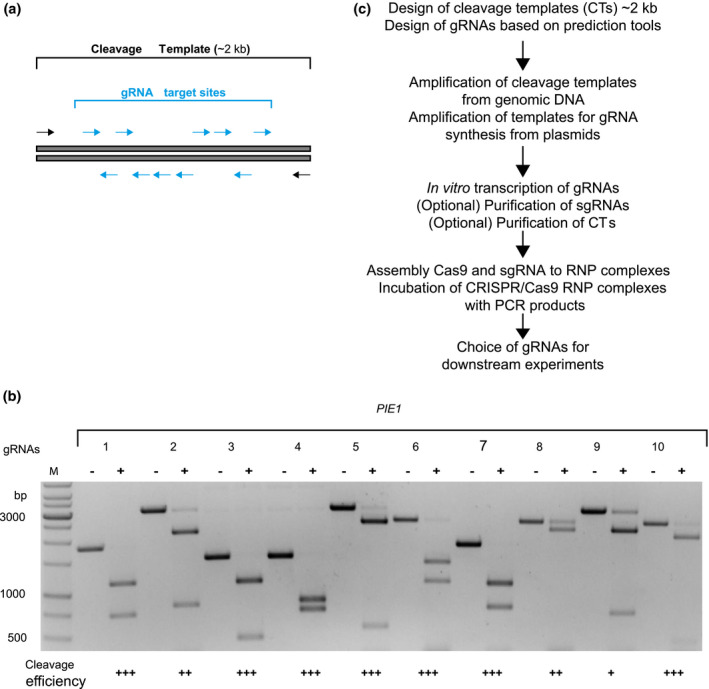
In vitro assay to estimate cleavage efficiency of sgRNA/Cas9 complexes on PCR‐amplified target DNA. (a) PCR products containing one or several sgRNA target sites (blue arrows) were amplified with flanking primers (black arrows) so that successful cleavage by a Cas9‐RNP would lead to fragments of different sizes. PCR‐generated templates should be 1–3 kb long to ensure sufficient resolution on a standard agarose gel. (b) Representative agarose gel after in vitro cleavage assays testing ten different sgRNAs (1–10) targeting the *PIE1* gene of *Arabidopsis thaliana*. PCR templates (of four different sizes, Table S1) incubated with recombinant Cas9 protein lacking a specific sgRNA (−) were not cleaved (−); addition of the sgRNA/Cas9 complex resulted in complete (+++), moderate (++), or poor (+) cleavage. Standard concentrations of Cas9, sgRNA, and cleavage template were 30 nM, 30 nM and 3 nM, respectively, incubation time was 1 hr at 37°C. M = 1 kb marker. (c) Overview of experimental workflow

Templates representing the genomic target sequence were amplified by PCR from Wt (Col‐0) genomic DNA. To allow fast and reliable evaluation of cleavage efficiency by agarose gel electrophoresis, primers were chosen to generate amplicons with fragments of distinguishable sizes after cleavage and electrophoresis (Figure 1a). PCR products with a size range between 1.5 and 3 kb were obtained using Q5 DNA polymerase (M0491, NEB), following manufacturer´s protocol. PCR products were then purified using DNA Clean and Concentrator (Zymo Research D4033), quantified with a Nanodrop ND1000 spectrophotometer (Thermo Fisher Scientific), and stored in aliquots at 4°C or −20°C.

### 2.7 Cas9‐cleavage assay of PCR products

Cas9 cleavage reactions (final volume 20 µl) were assembled as follows: 1 x Orange Buffer (O‐buffer; Thermo Fisher Scientific), 30 nM sgRNA, and 30 nM of recombinant Cas9‐NLS protein (M0386, NEB; in the present work provided by the IMP/IMBA/GMI Molecular Biology Core Facility), and ddH_2_O up to 15 µl were mixed and incubated for 10 min at 22°C to allow formation of the RNP. If not mentioned otherwise, 3 nM of cleavage template DNA (approximately 150 ng) were added and reaction volume brought to 20 µl with ddH_2_O if necessary. Reactions were incubated for 1 hr at 37°C and subsequently heat‐inactivated at 65°C for 15 min. Samples were either immediately analysed by electrophoresis on a 1% TAE‐agarose gel or stored at −20°C for later analysis.

For the genotyping cleavage assay, 150 ng of gDNA obtained from the pooled material by PCR as described above were cleaved with Cas9‐RNP as described for the individual amplicons, but incubation at 37°C was continued overnight. Heat inactivation was performed as mentioned, and 4 ng were used for PCR amplification with the corresponding primers (Table S1) and the following conditions: 96°C 2 min followed by 30 cycles of 96°C 30 s, 51°C 50 s, and 72°C 1 min followed by 72°C for 90 s, prior to analysis by gel electrophoresis.

### 2.8 Accession numbers

*ATM*: AT3G48190; *ATR*: AT5G40820; *RDR2:* AT4G11130; *RDR6:* AT3G49500; *AGO4* AT2G27040; *NRPD1a:* AT1G63020; *NRPE1:* AT2G40030; *ADH1:* AT1G77120; *CHLI1:* AT4G18480; *PIE1:* AT3G12810.

## 3 RESULTS

### 3.1 Test of sgRNA candidates based on in vitro cleavage efficiency

In contrast to genome editing in yeast or cultured mammalian cells, most knockout experiments in plants require passage of the mutation through the germ line. For recessive mutations, all alleles must be modified simultaneously. Considering long generation times and frequent polyploidy, the optimal choice of the sgRNAs prior to targeted knockout experiments in plants can save time, labor, and costs. We have therefore applied sgRNA design by in silico tools combined with a simple in vitro test for evaluating the cleavage efficiency of the ribonucleoprotein complex (RNP) prior to generating transgenic plants for the mutagenesis.

We chose eight different targets in the *Arabidopsis thaliana* genome and generated a list of potential sgRNAs for the genes *AGO4*, *NRPD1a*, *NRPE1*, *RDR2*, *RDR4*, *RDR6*, *CHLI1*, and *PIE1* with CHOPCHOP v2 (Labun et al., 2016), CCTOP (Stemmer et al., 2015), and CRISPR‐P v2.0 (Liu et al., 2017). Three sgRNAs each for seven of the targets, and 10 sgRNAs for *PIE1*, were compared for their predicted efficiency scores. This revealed congruency, but also discrepancy between the three tools (Table 1), which is plausible as they consider different datasets, gene features, base composition, and secondary structure.

**TABLE 1:** Comparison of sgRNA efficiency scores predicted by software tools compared to efficiency in the in vitro cleavage assay

gRNA		Efficiency of gRNA (predicted and estimated)			
Name	Sequence [5ʹ‐‐>3ʹ] PAM	CHOPCHOP	CCTOP	CRISPR‐P2	In vitro activity
PIE1_1	AGAGGCAGACGTTAATGCAG CGG	0.75	0.77	0.79	+++
PIE1_2	AGTAGGTCAAGATCATGGAG AGG	0.73	0.70	0.36	++
PIE1_3	TTAGTAATGGCGTCTAAAGG TGG	0.66	0.82	0.42	+++
PIE1_4	GAGGAAATGGAAGCTGGCGC AGG	0.70	0.67	0.25	+++
PIE1_5	AGACGTTATACCTCTGGAAG AGG	0.64	0.77	0.31	+++
PIE1_6	CGGCAACCAAGAATCAGGCG AGG	0.72	0.69	0.36	+++
PIE1_7	ATTTTGTCAACCCGAAGTGG GGG	0.71	0.66	0.46	+++
PIE1_8	CTTAGCAGAGCAATTGGAAG GGG	0.71	0.77	0.40	++
PIE1_9	ACTTTGGTAGTGCAAAGGAG CGG	0.70	0.71	0.89	+
PIE1_10	TGAAGCTAGAGAGAGGGAAG CGG	0.70	0.77	0.40	++
AGO4_1	TGGTTTCTTTGAGCAGGAGA TGG	0.62	0.55	0.30	+++
AGO4_2	ACATTTGGAGGTATAACTGG TGG	0.69	0.69	0.35	+++
AGO4_3	GTGGCTCTATTCTATGATGA TGG	0.44	0.64	0.14	+++
NRPD1a_1	TGAAGTTGTCTGTTTCTCAG TGG	0.62	0.80	0.08	++
NRPD1a_2	ATCGAGGTTAGAGTTCCCAC GGG	0.64	0.71	0.46	++
NRPD1a_3	TTTCGAATGTATTTACACCC CGG	0.50	0.65	0.54	+++
NRPE1_1	CGCCGTGTTTTTTTTTGGGG GGG	0.66	0.71	0.05	+
NRPE1_2	TTGACTGGGATGATTAATTG CGG	0.54	0.71	0.03	++
NRPE1_3	TCAGACATTTCAAGCACAGA AGG	0.63	0.58	0.05	+++
RDR2_1	TAACCCAAGAGAAAGAGAGA CGG	0.67	0.68	0.18	++
RDR2_2	AAACCATAAAGCTGTCACAG AGG	0.61	0.68	0.17	+++
RDR2_3	TGTGCCCTAGAAAAATGGGA TGG	0.65	0.60	0.82	+++
RDR6_1	TTATCAGCCTTATCATGTGG GGG	0.66	0.65	0.18	+++
RDR6_2	TGAAGGGGTTGATTTCCTGG TGG	0.67	0.84	0.17	++
RDR6_3	TGCAAGAGGAACGTGTGAGG TGG	0.72	0.65	0.05	+++
ADH1_1	GTCACACCGATGTTTACTTC TGG	0.34	0.64	0.03	+
ADH1_4	GGAGTGACTGATCTTCAGCC AGG	0.51	0.74	0.49	+++
ADH1_5	GCCTTTGATTACATGCTGAA GGG	0.46	0.58	0.48	+++
CHLI1_1	GATTTGCCTGTTTATAGTTT TGG	0.34	0.73	0.16	+++
CHLI1_6	GAACATCTTCTTCTGCAATC TGG	0.32	0.63	0.01	++
CHLI1_7	GAGTTTACTCAGCTTCTGAT TGG	0.36	0.64	0.40	+++
	Average	0.60 ± 0.13	0.69 ± 0.07	0.32 ± 0.23	

Note

Scores calculated for sgRNA sequences by three different tools are given in a range from 0 (poor) to 1 (high) efficiency. Ten sgRNAs targeting the *PIE1* gene and three sgRNAs each targeting the genes *RDR2*, *NRPE1*, *NRPD1a*, *RDR6*, *AGO4*, *ADH1*, and *CHLI1* of *Arabidopsis thaliana* were compared. The last column classifies the cleavage efficiency of each sgRNA, determined with the reconstituted RNP and the amplified target region in vitro as complete cleavage (+++, no uncut template left), partial cleavage (++, more cut fragments than uncut template), or poor cleavage (+, more uncut template than cut fragments, see Figure 1b and Figure S2).

We transcribed all sgRNAs in vitro with T7 polymerase and incubated them with recombinantly produced Cas9 protein to generate the RNPs. The RNPs were incubated with templates containing the intended cleavage sites in an asymmetric position of a PCR amplicon (Figure 1a, see Material and Methods for details). Samples were then analysed by gel electrophoresis (Figure 1b, Figure S2). The experimental procedure is summarized in Figure 1c. Among the ten sgRNAs targeting the *PIE1* gene, three (PIE1_2, PIE1_8, and PIE1_9) had limited efficiency and were discarded, as the templates were only partially cleaved (Figure 1b). In case of the other genes, we eliminated 8 of the 21 sgRNAs due to poor performance (Figure S2). However, all other seven PIE1 sgRNAs and 13 sgRNAs targeting the other genes displayed high cleavage efficiency.

While the experimentally assayed cleavage efficiency of the sgRNAs correlated with the in silico calculated scores in many cases, there were several exceptions in both directions: high‐scoring sequences with limited efficiency (e.g., PIE1_9), and low scoring sequences with complete cleavage (e.g., AGO4_3; CHLI1_7) (Table 1, Figure 1b, Figure S2). Thus, the in vitro cleavage assay helped to eliminate unsuitable, and confirm effective, sgRNAs.

### 3.2 Optimization of the in vitro cleavage assay with the Cas9‐sgRNA complex

To test the versatility and robustness of the in vitro cleavage assay, we performed the reaction in different commercially available buffers. Efficient sgRNAs showed cleavage across a wide range of conditions (Figure S3), although two of the buffers allowed only partial cleavage. Efficient sgRNAs showed partial cleavage even in water. For poorly performing sgRNAs, like ADH1_1 (Table 1 and Figure S3), neither a prolongation of the incubation time nor an excess of sgRNA could improve cleavage, whereas a complex with an efficient sgRNA, like ADH1_4, allowed complete cleavage in just five minutes and was also not affected by an sgRNA concentration beyond saturation of the complex (Figure S4a and b). While we used 37°C as the default incubation temperature, most plants, including Arabidopsis, are grown at lower temperatures. We, therefore, tested the in vitro cleavage efficiency at 21°C for three sgRNAs that performed well at 37°C on their respective templates. Two sgRNA/Cas9 assemblies (with (PIE1_4 and ADH1_4) cleaved equally well in both conditions, whereas the other (PIE1_3) was less efficient at the lower temperature, independent of the incubation time (Figure S4c).

While positive results from the in vitro assay do not guarantee in vivo cleavage when the target site is within a chromatin context, the assay can eliminate inefficient sgRNAs and identify those with good performance under the expected conditions, likely increasing the chance that they will also function in vivo. However, we reasoned that the robustness of this fast, simple, and cost‐effective sgRNA test protocol could be adapted also for downstream analysis of the mutagenesis by exploiting the efficient cleavage of remaining unmodified alleles and providing positive evidence for the presence of mutated alleles.

### 3.3 Efficient generation of knockout mutants with an optimized CRISPR/Cas9 vector

**FIGURE 2:**
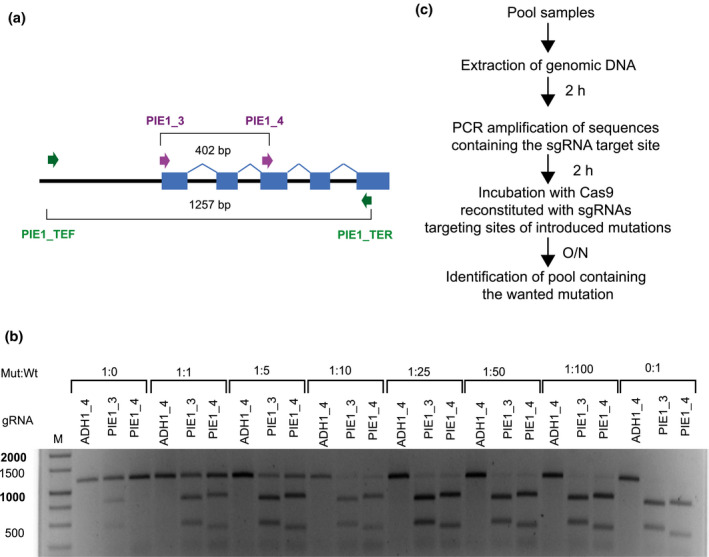
Determination of pool size for amplification of mutated alleles prior to in vitro cleavage with Cas9/sgRNA complex. (a) Location of primers and sgRNA target sites in the gene region. PIE1_3 and PIE1_4: binding sites for two sgRNAs used to mutate the *PIE1* gene of Arabidopsis. PIE_TEF_and PIE_TER: PCR primers flanking both sites. (b) gDNA extracted from pools of 50 individual seedlings, in different ratios between mutant line *atr2E3* and wild type (Wt), was amplified with the primers PIE_TEF and PIE_TER and the amplicons digested overnight with Cas9‐RNPs reconstituted with PIE1_3, PIE4, or ADH1_4 (control) sgRNA, prior to gel electrophoresis. (c) Overview of experimental workflow

Although introducing reconstituted Cas9‐sgRNA complexes into cells (without the genes encoding the components) is a convenient procedure (Park & Choe, 2019), this approach is often not successful in generating mutations that are passed through the germline. In such cases, coding sequences for Cas9 and sgRNAs must be introduced into plant cells by transfection or transformation. Afterwards, they should be separated from the mutation as soon as possible to reduce the risk of off‐target mutations upon further propagation. The design of suitable vectors requires specific consideration for different species and cannot be generalized. Here, we explain a strategy chosen to mutagenize a specific Arabidopsis gene as a proof of concept. Based on the pDe‐CAS9 backbone (Fauser et al., 2014), we generated vectors containing the codon‐optimized *SpCas9* gene under the control of the promoter EC1.2 (Wang et al., 2015) that drives expression specifically in the egg cell, the haploid stage of female gametogenesis. This creates the possibility to cut DNA not only at the maternal allele but also at the incoming paternal copy after fertilization, so that homozygous mutant alleles are possible already in the next generation. We also included a modified cassette with the Oleosin::GFP selection marker (Shimada et al., 2010) that confers seed‐specific GFP expression. This enables fast selection of transgenic seeds and screening for transgene‐free, Cas9‐negative segregants in the generation after mutagenesis. Mutagenic efficiency was further increased using the Arabidopsis *U6‐26* promoter to express a multiplexed array of several sgRNAs, separated by the endogenous tRNA^gly^ sequence. This generated mature single sgRNAs through subsequent RNA processing (Xie et al., 2015). A map of the vector pDEECO is shown in Figure S1. To develop the genotyping protocol for Cas9‐generated mutations, we decided to mutate the Arabidopsis *PIE1* gene. This >9000 bp long gene encodes a subunit of the SRW1 chromatin remodelling complex, and recessive homozygous mutations result in early flowering, serrated leaves, and stunted growth, phenotypes which are easily distinguishable already in young plants (Noh & Amasino, 2003). Another reason to choose this target gene was the reduced fertility of *pie1* mutants, which make conventional crossings with other lines difficult and laborious. The well‐performing sgRNAs PIE1_3 and PIE1_4 (Table 1, Figure 1b, Figures S3 and S4) are located 402 bp apart near the 5’ end of the gene, and we expected that single site mutations at either site as well as a deletion between them would cause a complete loss‐of‐function (Figure 2a). We inserted sgRNAs PIE1_3 and PIE1_4 into the pDEECO vector and transformed Col‐0 plants. The same construct was also introduced into *atm* and *atr* mutant plants, which have defects in the DNA damage response (Culligan, Robertson, Foreman, Doerner, & Britt, 2006) and might modify the repair mode at the cleavage sites. GFP‐expressing seeds from the transformed plants were grown to maturity to generate seeds. The plants from the T2 generation were grown and scored for the characteristic *pie1* mutant phenotype, which would indicate mutations at both alleles of the diploid plant. We calculated the frequency of mutant phenotypes from nine independent T2 lines (Table 2) to be 28.6% ± 19.0%, with only minor differences between wild‐type (Wt) and *atm* or *atr* mutants. The high variation between different independent transgenic lines could result from positional effects that modulate the CRISPR/Cas9 or sgRNA expression at their respective insertion sites. However, many mutations do not cause an easily scorable phenotype as in case of the *PIE1* target gene, and mutation rates are often much lower than achieved in Arabidopsis. This makes their identification time‐, labor‐, and resource‐consuming, thus requiring screening of a large number of plants. Therefore, we have used the spectrum of CRISPR/Cas9‐generated *pie1* mutants as material to test whether the in vitro assay can be used to genotype material potentially containing mutant alleles. We selected GFP‐negative T2 seeds from plants with the mutant phenotype to eliminate the CRISPR/Cas9 T‐DNA module as described above.

**TABLE 2:** Frequency of progeny with mutant phenotype after Cas9 mutagenesis

Genotype	Line	*pie1* phenotype	Number of plants	%
Col‐0	2	1	45	2.2
Col‐0	3	8	45	17.8
Col‐0	4	20	40	50.0
*atm*	3	12	35	34.3
*atm*	4	12	37	32.4
*atm*	6	17	28	60.7
*atr*	2	13	33	39.4
*atr*	3	11	36	30.6
*atr*	4	3	40	7.5
All		97	339	28.6
Col‐0	—	0	18	0
*pie1‐5*	—	17	17	100

Note

Offspring of Arabidopsis wildtype (Col‐0) or mutants impaired in DNA repair (*atm*, *atr*) were transformed with pDEECO::PIE1KO targeting the *PIE1* gene, and progeny of three independent transformants each were grown for two weeks under long day conditions. The presence of serrated leaves and stunted growth phenocopied known *pie1‐5* knockout mutants (Noh & Amasino, 2003) and were therefore considered to indicate biallelic mutation in the *PIE1* gene due to CRISPR/Cas9 activity. Col‐0 and *pie1‐5* plants were grown under the same conditions as controls.

### 3.4 Modifying the in vitro cleavage assay with the Cas9‐sgRNA complex for screening mutant alleles in pools

Mutants bearing large deletions are easily detected by PCR on pooled genomic DNA with primers outside both sgRNA target sequences, but mutations or indels at only one or both target sites can be small and undetectable by electrophoresis of amplicons. However, such mutations disrupt the target sequence, conferring resistance to cleavage by the Cas9‐sgRNA complex with which they had been generated. We wanted to determine the degree to which a single base mutation inhibited cleavage and the sensitivity of the assay in pools of multiple individuals. Among the lines listed in Table 2, *atr pie1* 2E3 was homozygous for a 1 bp insertion at the PIE1_3 site and a 4 bp deletion at the PIE1_4 target site, probably originating from consecutive cleavage and independent repair at each site. We prepared pools with different ratios (1:1 to 1:100, in addition to pure samples) of individual cotyledons from mutant and Wt seedlings, extracted genomic DNA from the pools, and amplified the region containing both potential mutation sites by PCR. The amplicons were incubated overnight with Cas9 RNPs containing either of the two sgRNAs used for the mutagenesis. Samples from pools with only Wt plants were completely cleaved, while a full‐size band was detectable in the mutant/Wt range up to 1:25 (Figure 2b). Alternatively, genomic DNA from the pools can be cleaved prior to amplification with the flanking primers, reducing the analysis to that for the full‐size band (Figure S5). In both procedures, no unspecific cleavage was observed with a control sgRNA, but slight differences between the two mutation sites became evident: the 1 bp mutation at the PIE1_3 site allows minor, but detectable cleavage in pure mutant pools (Figure 2b), congruent with less sensitivity when the genomic DNA was incubated with the RNP before amplification (Figure S5). In pure Wt samples, a faint band amplified from undigested template suggests that the genomic DNA might not be as cleavable as the PCR amplicons. Thus, this control should always be included. However, in both protocols, generating positive evidence for even minimally mutated alleles in pools of > 10 can substantially increase speed and throughput of the mutant screen.

**FIGURE 3:**
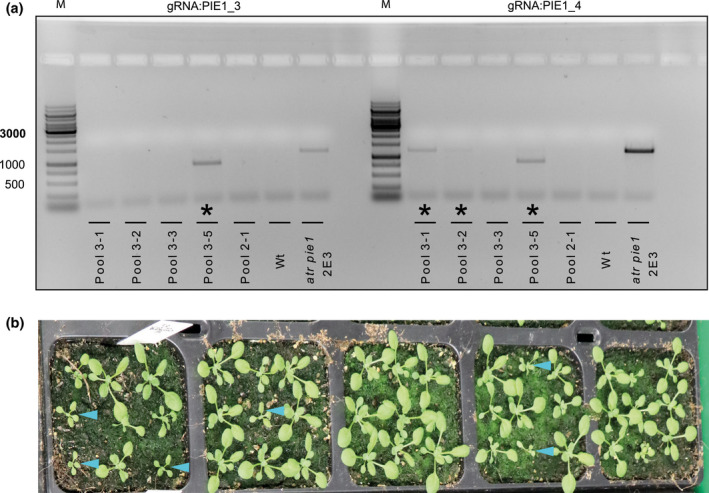
Validation of Cas9‐RNP genotyping to identify mutants in pools of candidates. (a) Progeny from plants transformed with pDEECO::PIE1KO containing multiplexed PIE1_3 and PIE1_4 sgRNAs were grown, nine cotyledons each from five independent populations pooled and used to extract genomic DNA. These samples were incubated overnight with Cas9‐RNPs reconstituted with either PIE1_3 or PIE1_4 sgRNAs, and aliquots of 4 ng amplified with primers flanking both sites. * marks pools with amplified bands, indicating the presence of mutated alleles. Wt (Col‐0): wild‐type control for complete cleavage, *atr pie1* 2E3: a previously characterized line with single mutations at both sites as positive control. M = size marker. (b) Seedlings from the five populations (in the same order as in (a)) were grown on soil. Blue arrows in pool 3–1, 3–2, and 3–5 indicate plants with the *pie1* phenotype (serrated leaves and stunted growth), indicating homozygous mutant alleles, frequent biallelic mutations, and congruency with the genotyping assay in (a)

We applied the Cas9 cleavage‐based amplification of mutant alleles on five pools of cotyledons, each containing nine individual T2 plants that had been mutagenized with the two multiplexed sgRNAs PIE1_3 and PIE1_4 but not pre‐selected for the mutant phenotype. Genomic DNA from each pool was incubated overnight with Cas9‐RNP reconstituted with either sgRNA. Four ng of DNA from each reaction was then used for PCR with primers spanning both sgRNA target sites. No amplification was detected in Wt controls, confirming complete cleavage at both sgRNA binding sites, whereas an amplicon of the expected size was obtained with DNA from the previously identified and described mutant *atr pie1* 2E3 as positive control (Figure 3a). PCR products in pools 3–1, 3–2, and 3–5 indicated mutations, three out of five at the PIE1_4 site and one at the PIE1_3 site (Figure 3a). The 850 bp amplicon in pool 3–5 suggested that cleavage at both sites resulted in a deletion (Figure 2a). Amplification of the full‐length band in pools 3–1 and 3–2 indicated resistance to cleavage only with Cas9/PIE1_4, suggesting mutations only at this target site (Figure 3). Growing the T2 populations on soil confirmed the presence of mutated alleles, as the characteristic phenotype was clearly segregating (Figure 3b). Therefore, the protocol described here can score for the presence of even minor mutations in larger populations, reducing the number of required experiments and re‐using material that had been designed for the mutagenesis procedure.

### 3.5 The in vitro cleavage assay with the Cas9‐sgRNA complex for fast and reliable mutation screening

**FIGURE 4:**
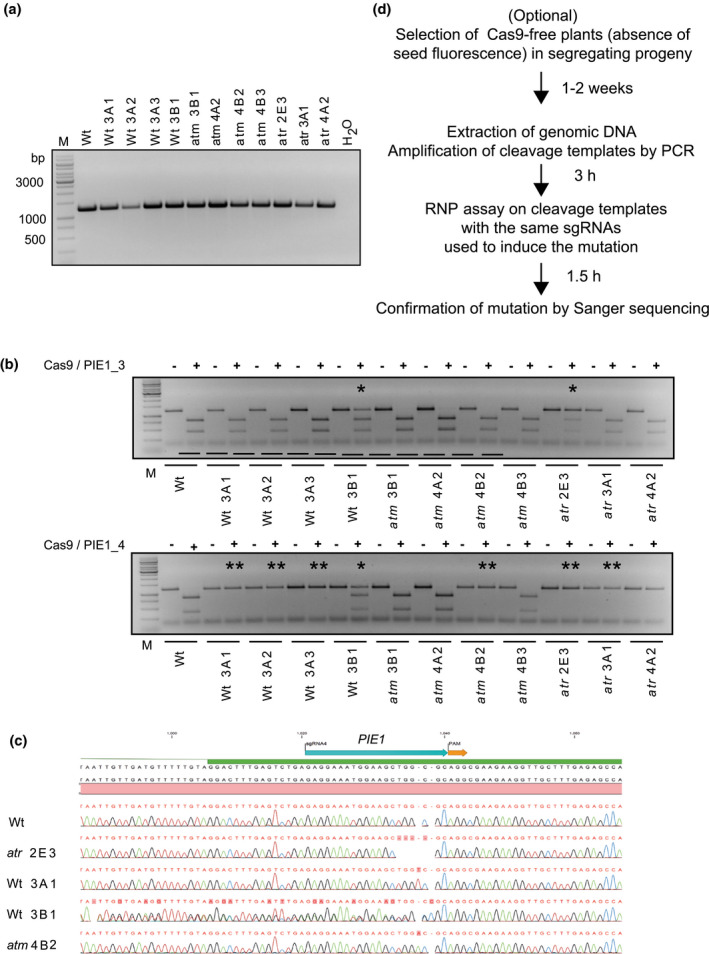
Validation of Cas9‐RNP genotyping to characterize individual mutations. (a) GFP‐negative seeds in progeny of Cas9‐mutagenized plants (Table 2), indicating loss of the Cas9 transgene by segregation, were grown and genomic DNA was prepared from individual plants. This was amplified with primers flanking the mutation target sites. (b) Amplicons from (A) untreated (−) or after in vitro incubation (+) with Cas9 reconstituted with PIE1_3 (top) or PIE1_4 (bottom). **marks non‐cleaved amplicons from homozygous mutant plants, * marks partially cleaved amplicons from heterozygous plants. Wt: Col‐0 control for complete cleavage. (c) Non‐cleaved amplicons from Cas9‐RNP‐genotyped mutations in (b) were subjected to Sanger sequencing, confirming different independent mutations and homo‐ and heterozygosity. (d) Overview of experimental workflow

Individuals from pools with DNA not cleaved by the RNP can be further characterized with the same principle, by generating the amplicon around the target site (Figure 4a) and incubating it with the reconstituted RNP according to the protocol described. Full‐length amplicons indicate at least one mutated allele, whereas cleavage affects the unmodified Wt sequences (Figure 4b). Equal presentation of cleaved and non‐cleaved bands (e.g., Wt 3B1 in the lower panel of Figure 4b) indicates co‐existence of Wt and mutant allele (heterozygosity), while complete protection (e.g., Wt 3A1) suggests homozygosity with mutated alleles only. The non‐cleaved amplicons can be immediately used for Sanger sequencing, and we obtained perfect congruency between the genotyping results and the sequence analysis (examples in Figure 4c). The workflow of this fast protocol is shown in Figure 4d. It takes only a few hours and can be scaled up for high‐throughput procedures, as genomic DNA from rapid extraction protocols is suitable and PCR amplicons do not need any additional purification steps.

### 3.6 Validation of the protocol for a different target gene

We have further applied this procedure to genotype mutations in a gene that does not cause morphological defects or other easily scorable changes. The gene encoding RNA‐dependent RNA polymerase 2 is essential for epigenetic regulation in Arabidopsis. Mutants cannot produce small RNAs, but are otherwise phenotypically indistinguishable from Wt plants (Willmann, Endres, Cook, & Gregory, 2011). After transgenesis with pDEECO::RDR2KO (containing sgRNA RDR2_3, Table S1), plants were analysed and pre‐screened in pools as described for the PIE1 mutants, but reconstituting the Cas9 protein with sgRDR2_3. Amplicons spanning the mutation sites were partially or fully resistant to cleavage at 21°C and 37°C in all but two plants, indicating the presence of mutant alleles (Figure S6a). Sanger sequencing of the amplicons revealed several independent insertions and deletions at the sgRNA site and confirmed the unmodified Wt sequence in the two plants in which the amplicon was completely cleaved (Figure S6b). In summary, the in vitro cleavage assay with Cas9‐RNPs containing efficient sgRNAs can be used as a quick, easy, and cost‐efficient assay to identify and characterize CRISPR‐generated genomic mutations.

## 4 DISCUSSION

We described an in vitro assay for simple and resource‐saving screens of mutated alleles, by “recycling” the components of the in vitro cleavage assay. We have explored the suitability and range of application with a proof of concept by genotyping mutations in two genes of the model plant Arabidopsis, but the principle is universally transferable. The procedure requires no special equipment and only those components that are anyway necessary and had been used for CRISPR/Cas9 mutagenesis. Nothing beyond standard cloning procedures, DNA extraction, in vitro transcription of sgRNAs, PCR amplification, and gel electrophoresis is needed. All components are commercially available or can even be produced in the lab at low cost (e.g., Rajagopalan, Kagale, Bhowmik, & Song, 2018).

Despite the exponentially growing application of CRISPR mutagenesis, there are still cases where the success rate is low, and low sgRNA efficiency can be one of the limiting factors. Software tools are being trained to make better predictions of successful sgRNAs based on experience in different systems, mostly animal and human and the requirement for a suitable protospacer adjacent motif (PAM) close to the wanted mutation is being reduced by the growing range of Cas variants with different PAM requirements (Zhang, Malzahn, Sretenovic, & Qi, 2019). However, experimental validation of cleavage efficiency remains an important first step, especially for germline mutagenesis of plants with long generation times or challenging transformation procedures. Multiple assays built on different principles to test different sgRNAs have been described, including the surveyor nuclease assay, cleaving heteroduplex DNA between non‐modified and modified sequences (Guschin et al., 2010; Liu, Homma, et al., 2016) or protocols based on chemiluminescence (Liu, Yu, et al., 2016) or isothermal amplification (Zhang et al., 2016). These are reliable and widely applied, but need individual sample treatment, laborious processes, extra material, or equipment. Assays similar to the one applied in our work have been described (e.g., Cong et al., 2013; Dang et al., 2015; Grainger et al., 2017; Matson et al., 2019) and are supported with commercial tools (e.g., www.takarabio.com). While identifying efficient sgRNAs can certainly reduce the risk of failure and save labor and costs, in vitro assays with plain DNA cannot predict the accessibility of different target sites for the RNPs in vivo. This could still be a serious limitation for absolute and relative numbers of independent mutation events, for which likely no universal solution is available. Therefore, efficient screening for mutant alleles becomes pivotal when low mutation rates, or time‐consuming, expensive, laborious cultivation of individuals represent major limiting factors. Controlled modifications of complex metabolic or developmental pathways for accelerated breeding or domestication of wild species requires mutations at several genes at the same time. Also here, simplification, acceleration, or cost reduction by screening a large number of individuals at multiple sequences and encompassing heterozygous genotypes can have substantial impact.

Genotyping CRISPR/Cas9‐generated mutations has been performed utilizing heteroduplex DNA fragments formed between modified and non‐modified DNA and their different mobility during electrophoresis (e.g., in zebrafish by Sorlien et al., 2018). Protocols using sgRNA in connection with Cas9 for a restriction enzyme‐like genotyping assay similar to the one described here have also been reported (Kim et al., 2014; Liang et al., 2018; Zhou, Peng, Zhang, & Li, 2018), but with a time‐consuming protocol limited to short PCR fragments which renders it difficult to test several sgRNAs with the same amplified target. Next generation sequencing of amplicons derived from CRISPR/Cas9‐edited populations can be used to screen for desired mutations (Fernández et al., 2020), but this requires costly library preparation and sequence data analysis and cannot be routinely used in every lab. Our design, with its proven robustness in different buffers, allows for a versatile choice of template fragments, is suitable for testing multiple sgRNA sites at the same time, and can be combined with other enzymatic reactions. We demonstrated that the genotyping protocol works with genomic DNA prior to amplification, making it suitable for fast upscaling of screening multiple targets from the same samples and application of quantitative methods as qPCR for testing minor differences. Lastly, we improved the CRISPR/Cas9 genotyping in pools of genomic DNA, proving the sensitivity and the high‐throughput capability of our workflow. This reduces labor and costs for pre‐screening CRISPR/Cas9‐induced mutations especially in case of low mutation rates. The easy detection of cleavage products by electrophoresis allows for a simple and fast read‐out that already contains information about zygosity of the mutation. This is particularly valuable in case of a polyploid genome, to rapidly identify individuals in which all alleles were modified.

This cleavage assay, modified to detect mutated target DNA molecules among many more sensitive ones within pools of genomic DNA from several individuals, provides an efficient and cost‐effective screen to further overcome limitations in CRISPR‐based mutagenesis. The assay can reduce sample numbers, amount of PCR reagents, and labor without affecting its robustness. The genotyping assay works in different buffers and with DNA from most standard preparation protocols. If only specific mutant alleles are wanted, such as single nucleotide deletion/insertions or in‐frame mutations, non‐cleaved fragments amplified in pools could be cloned into E. coli for Sanger sequencing to identify whether the pool contains the desired mutation. Using the same components as in the initial sgRNA test for genotyping, we utilize the high sequence specificity of in vitro cleavage by Cas9‐RNPs twice, without additional costs and resources. Upscaling the preparation of sgRNAs for the initial cleavage assay is easy and recommended, as aliquots of the tested batches are later available for the genotyping.

In summary, using the same components and protocols to score for successful target cleavage prior to mutagenesis and for identifying mutated alleles afterwards can make generating genome‐edited plants faster and more efficient, especially in species with long generation times, less established transformation protocols, or polyploid genomes. Since it does not require additional reagents, it can be used as routine procedure suitable for high‐throughput experiments, teaching courses and outreach demonstrations. As the Cas toolbox is rapidly expanding (Pickar‐Oliver & Gersbach, 2019; Zhang et al., 2019), we expect that our in vitro assay can be extended and modified in many different directions.

## CONFLICT OF INTEREST

The authors declare no conflict of interest.

## AUTHORS CONTRIBUTIONS

H.B. and M.D. designed and carried out the experiments. All authors discussed the results and wrote the manuscript.

## Supporting information

Supplementary Material
