# Supplementary material for: Versatile in vitro assay to recognize Cas9‐induced mutations
Source: Plant Direct. 2020 Sep 28;4(9):e00269. doi: 10.1002/pld3.269 (PMC7522499; doi:10.1002/pld3.269)
Supplement: Supplementary file 1 — Supplementary Material [file PLD3-4-e00269-s001.pdf]

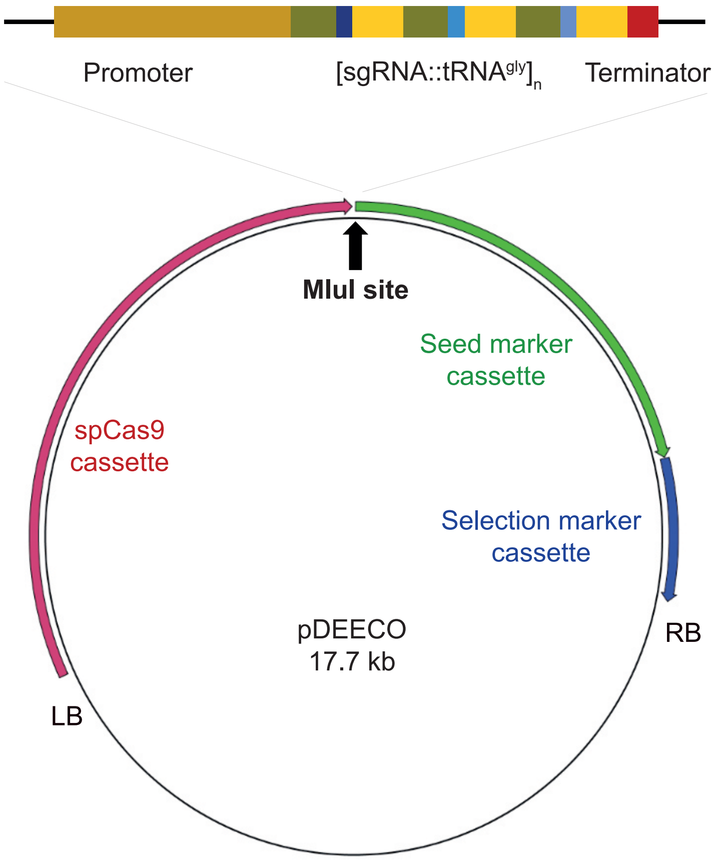

Bente et al. Supplemental Figure 1

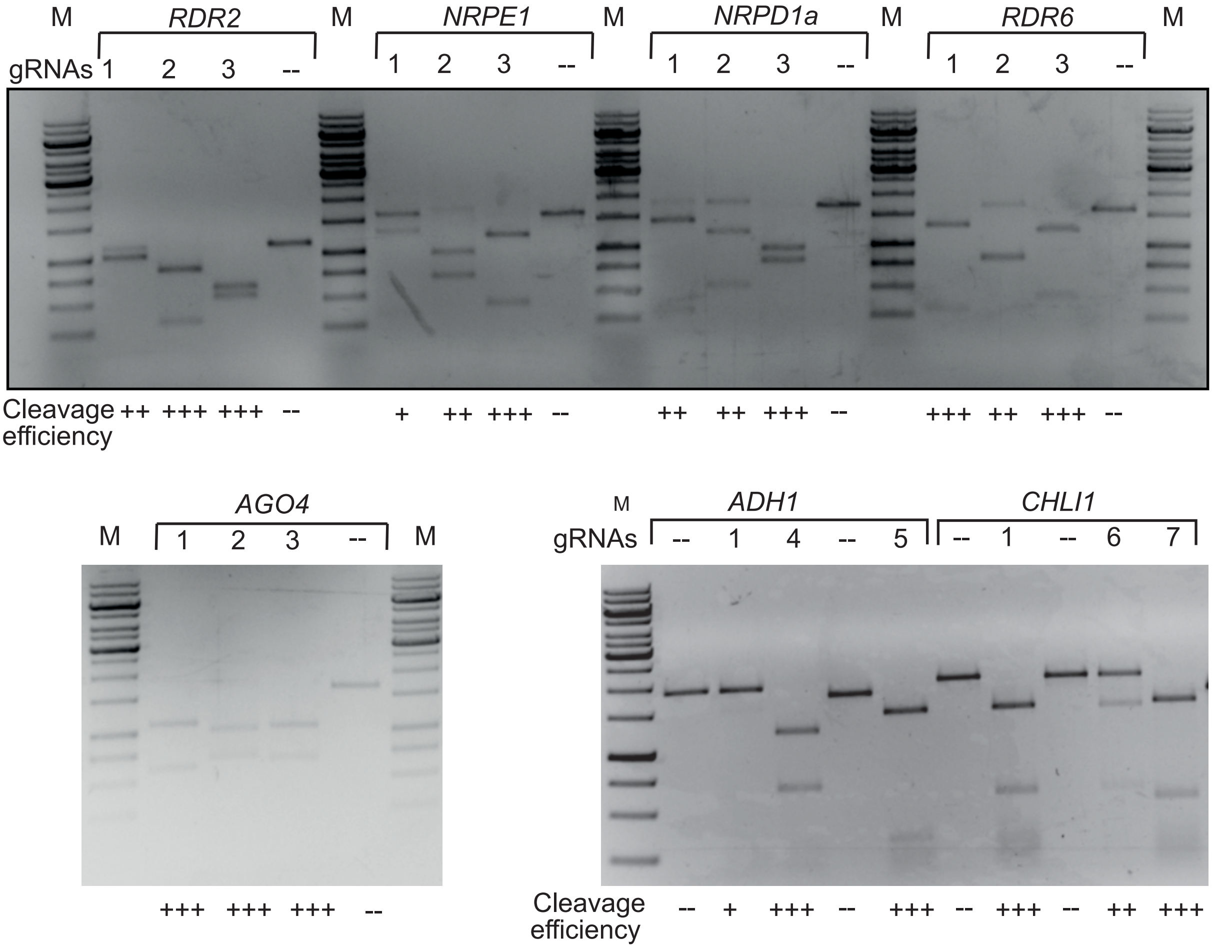

Bente et al. Supplemental Figure S2

A

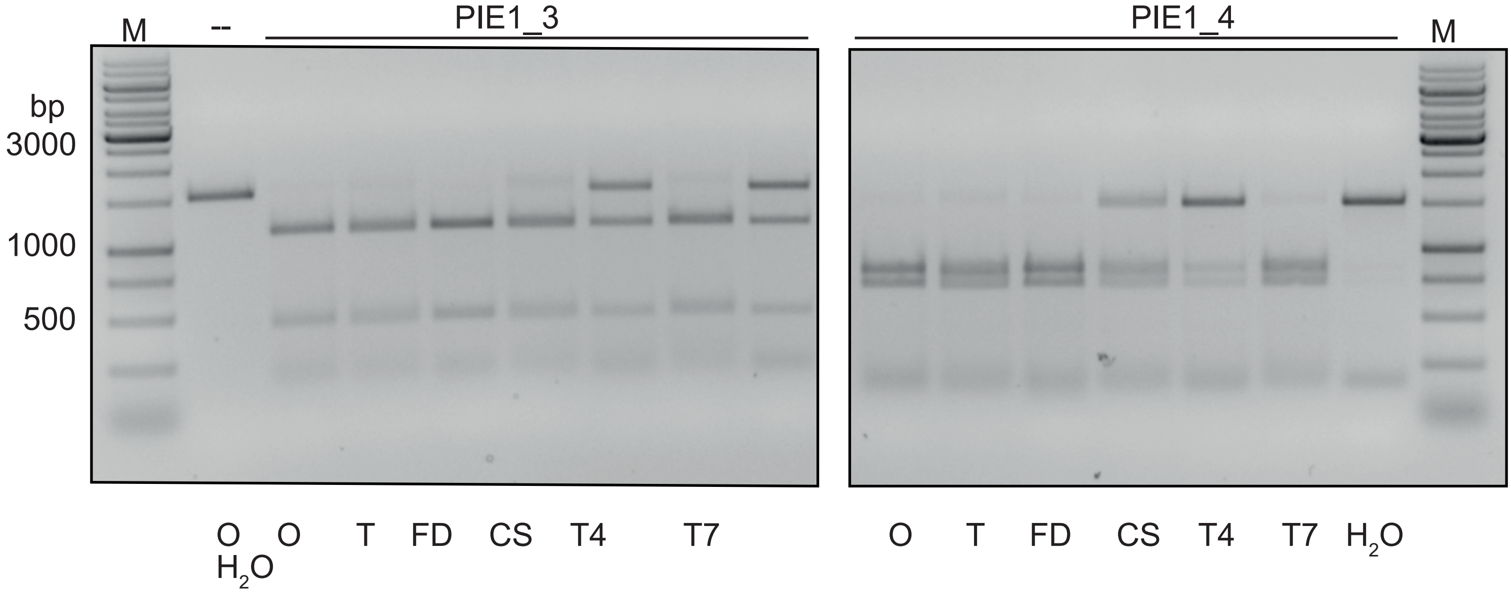

B

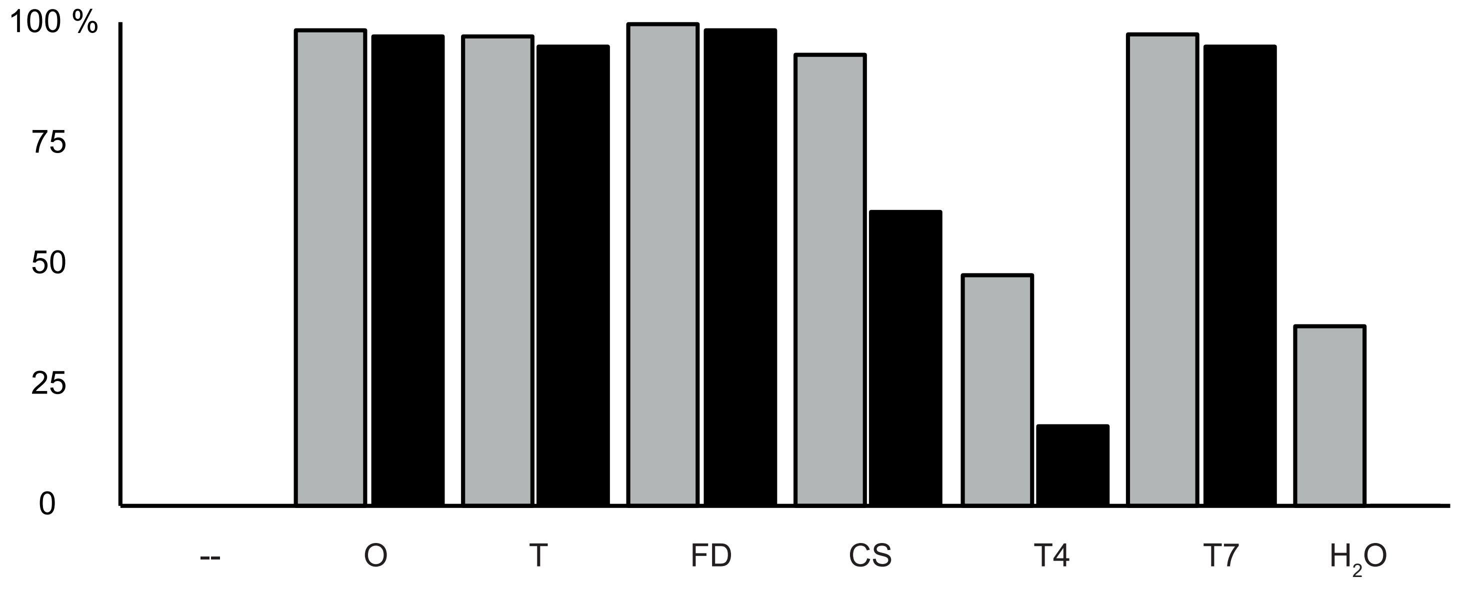

Bente et al. Supplemental Figure 3

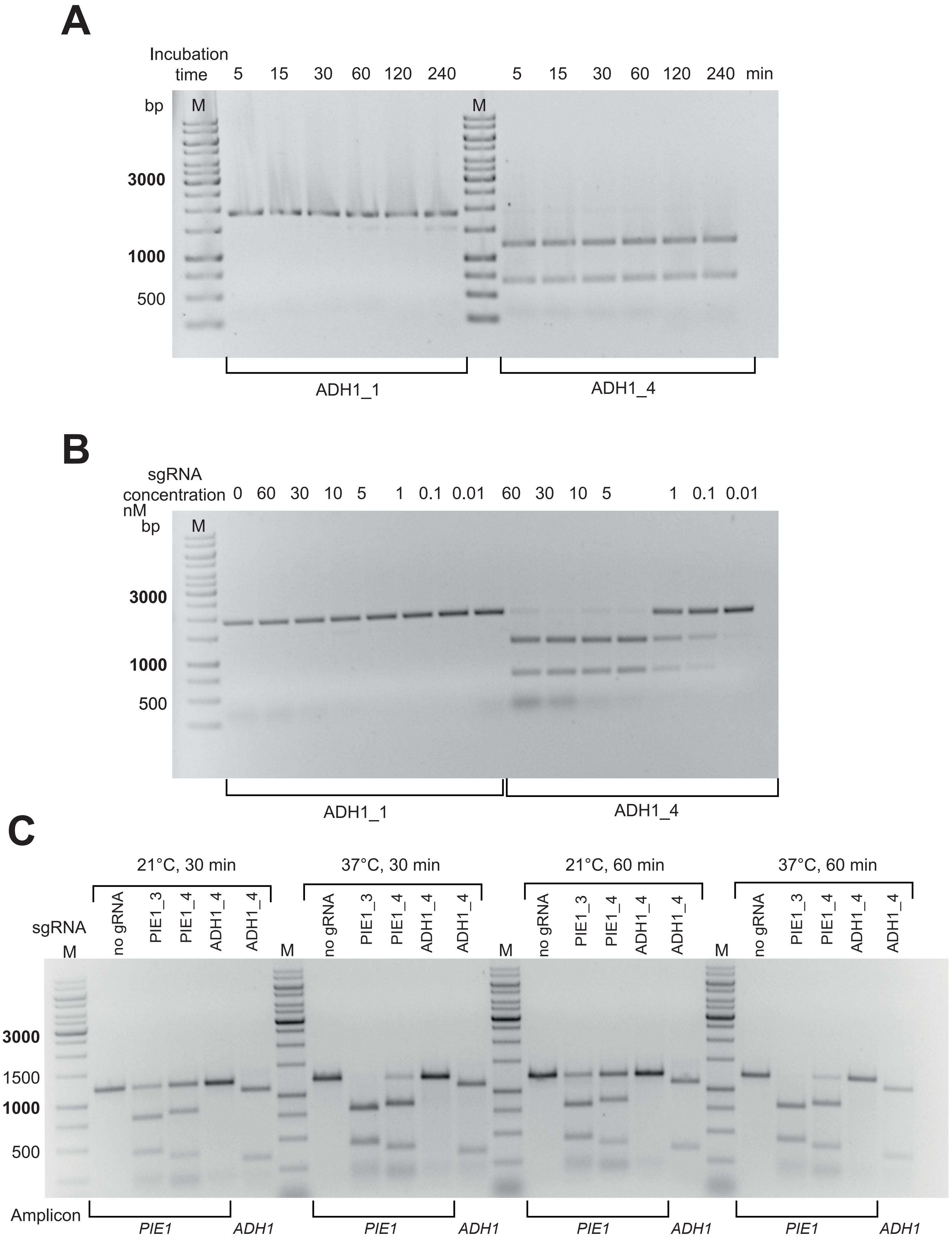

Bente et al. Supplemental Figure S4

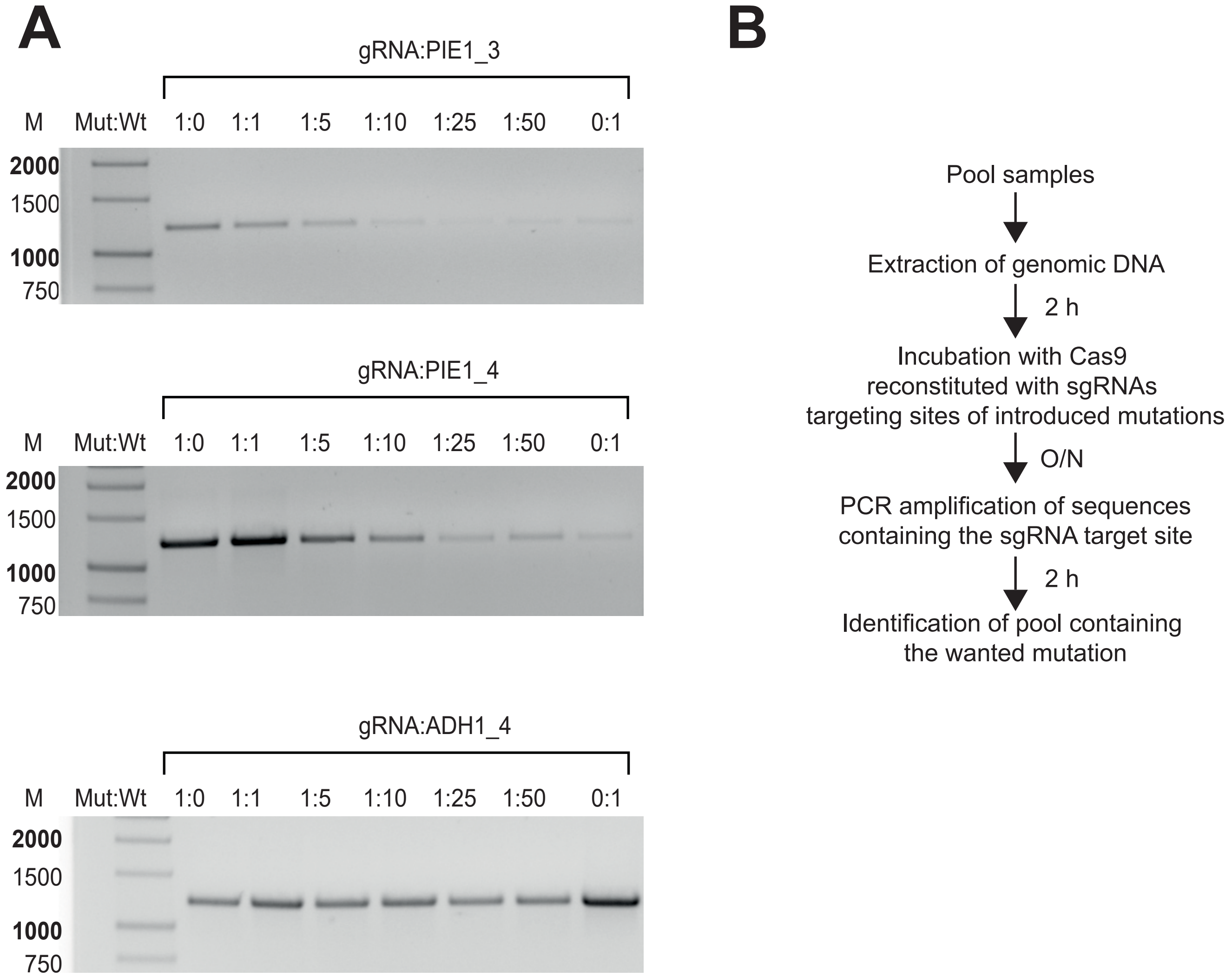

Bente et al. Supplemental Figure S5

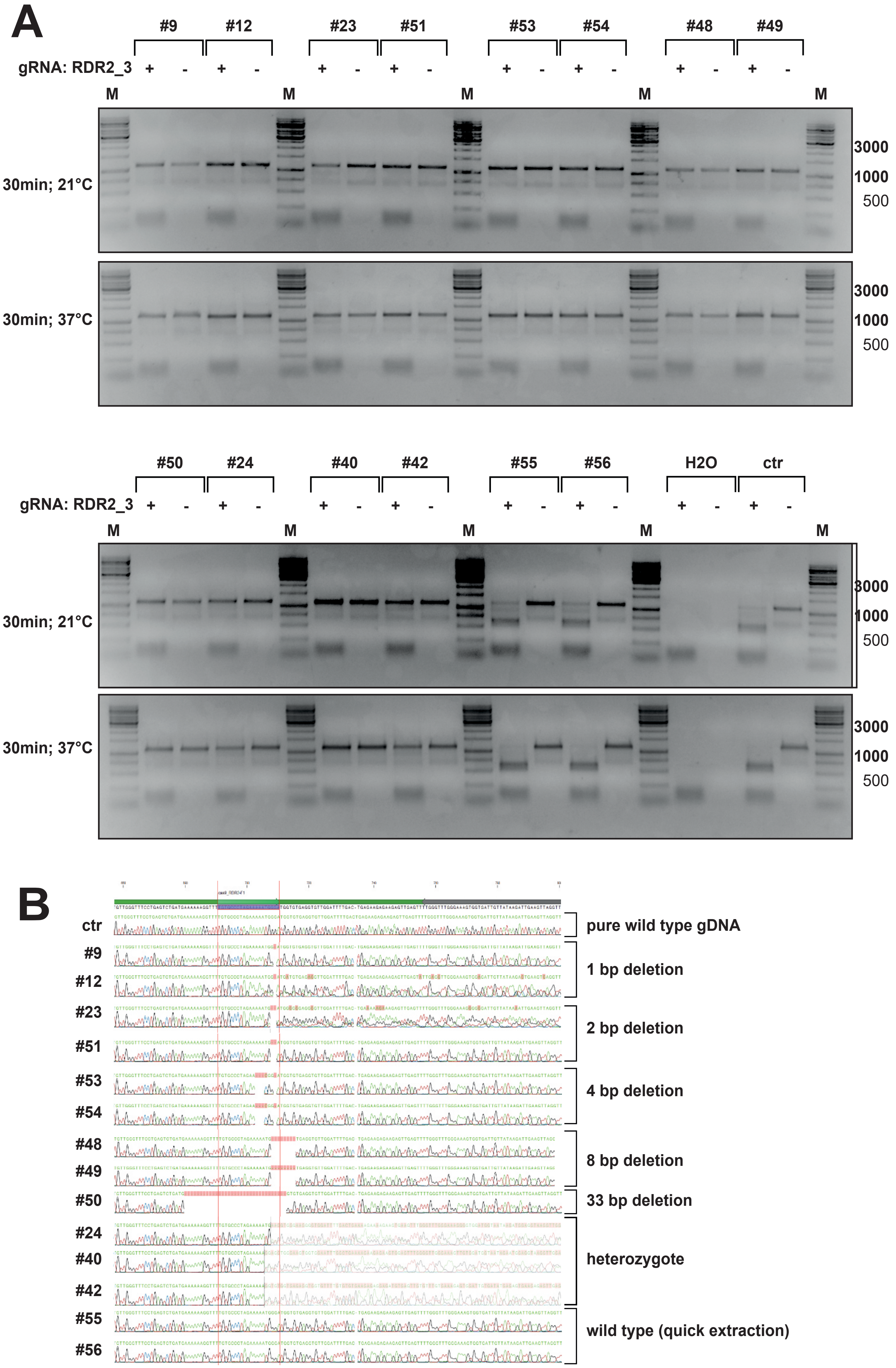

Bente et al. Supplemental Figure S6

Supplementary Table S1: Oligonucleotides and PCR annealing temperatures

Name	Sequence (5'-->3')	Annealing temperature [°C]	Purpose
**sgRNAs**			
ADH1_1	GCGGCCTCTAATACGACTCACTATAGGaagtaaacatcggtgtgacGTTTTAGAGCTATGCTGAAA	61	sgRNA targeting *ADH1*
ADH1_4	GCGGCCTCTAATACGACTCACTATAGGgagtgactgatcttcagccGTTTTAGAGCTATGCTGAAA	61	sgRNA targeting *ADH1*
ADH1_5	GCGGCCTCTAATACGACTCACTATAGGcctttgattacatgctgaaGTTTTAGAGCTATGCTGAAA	61	sgRNA targeting *ADH1*
AGO4_1	GCGGCCTCTAATACGACTCACTATAGGtggtttctttgagcaggagAGTTTTAGAGCTATGCTGAAA	61	sgRNA targeting *AGO4*
AGO4_2	GCGGCCTCTAATACGACTCACTATAGGacatttggaggtataactggGTTTTAGAGCTATGCTGAAA	61	sgRNA targeting *AGO4*
AGO4_3	GCGGCCTCTAATACGACTCACTATAGGgtggctctattctatgatgaGTTTTAGAGCTATGCTGAAA	61	sgRNA targeting *AGO4*
CHLI1_1	GCGGCCTCTAATACGACTCACTATAGGatttgcctgtttataGTTTGTTTTAGAGCTATGCTGAAA	61	sgRNA targeting *CHLI1*
CHLI1_6	GCGGCCTCTAATACGACTCACTATAGGaacatcttcttctgcaatcGTTTTAGAGCTATGCTGAAA	61	sgRNA targeting *CHLI1*
CHLI1_7	GCGGCCTCTAATACGACTCACTATAGGagtttactcagcttctgatGTTTTAGAGCTATGCTGAAA	61	sgRNA targeting *CHLI1*
NRPD1a_1	GCGGCCTCTAATACGACTCACTATAGGtgaagttgtctgtttctcagGTTTTAGAGCTATGCTGAAA	61	sgRNA targeting *NRPD1a*
NRPD1a_2	GCGGCCTCTAATACGACTCACTATAGGatcgaggttagagttcccacGTTTTAGAGCTATGCTGAAA	61	sgRNA targeting *NRPD1a*
NRPD1a_3	GCGGCCTCTAATACGACTCACTATAGGtttcgaatgtatttacacccGTTTTAGAGCTATGCTGAAA	61	sgRNA targeting *NRPD1a*
NRPE_1	GCGGCCTCTAATACGACTCACTATAGGcgccgtgtttttttttggggGTTTTAGAGCTATGCTGAAA	61	sgRNA targeting *NRPE1*
NRPE_2	GCGGCCTCTAATACGACTCACTATAGGttgactgggatgattaattgGTTTTAGAGCTATGCTGAAA	61	sgRNA targeting *NRPE1*
NRPE_3	GCGGCCTCTAATACGACTCACTATAGGtcagacatttcaagcacagaGTTTTAGAGCTATGCTGAAA	61	sgRNA targeting *NRPE1*
PIE1_1	GCGGCCTCTAATACGACTCACTATAGGagaggcagacgttaatgcagGTTTTAGAGCTATGCTGAAA	61	sgRNA targeting *PIE1*
PIE1_2	GCGGCCTCTAATACGACTCACTATAGGagtaggtcaagatcatggagGTTTTAGAGCTATGCTGAAA	61	sgRNA targeting *PIE1*
PIE1_3	GCGGCCTCTAATACGACTCACTATAGGttagtaatggcgtctaaaggGTTTTAGAGCTATGCTGAAA	61	sgRNA targeting *PIE1*
PIE1_4	GCGGCCTCTAATACGACTCACTATAGGgaggaaatggaagctggcgcGTTTTAGAGCTATGCTGAAA	58	sgRNA targeting *PIE1*
PIE1_5	GCGGCCTCTAATACGACTCACTATAGGagacgttatacctctggaagGTTTTAGAGCTATGCTGAAA	58	sgRNA targeting *PIE1*
PIE1_6	GCGGCCTCTAATACGACTCACTATAGGcggcaaccaagaatcaggcgGTTTTAGAGCTATGCTGAAA	61	sgRNA targeting *PIE1*
PIE1_7	GCGGCCTCTAATACGACTCACTATAGGattttgtcaacccgaagtggGTTTTAGAGCTATGCTGAAA	61	sgRNA targeting *PIE1*
PIE1_8	GCGGCCTCTAATACGACTCACTATAGGcttagcagagcaattggaagGTTTTAGAGCTATGCTGAAA	61	sgRNA targeting *PIE1*
PIE1_9	GCGGCCTCTAATACGACTCACTATAGGactttggtagtgcaaaggagGTTTTAGAGCTATGCTGAAA	61	sgRNA targeting *PIE1*
PIE1_10	GCGGCCTCTAATACGACTCACTATAGGtgaagctagagagagggaagGTTTTAGAGCTATGCTGAAA	61	sgRNA targeting *PIE1*
RDR2_1	GCGGCCTCTAATACGACTCACTATAGGtaacccaagagaaagagagaGTTTTAGAGCTATGCTGAAA	61	sgRNA targeting *RDR2*
RDR2_2	GCGGCCTCTAATACGACTCACTATAGGtgtgccctagaaaaatgggaGTTTTAGAGCTATGCTGAAA	61	sgRNA targeting *RDR2*
RDR2_3	GCGGCCTCTAATACGACTCACTATAGGaaaccataaagctgtcacagGTTTTAGAGCTATGCTGAAA	61	sgRNA targeting *RDR2*
RDR6_1	GCGGCCTCTAATACGACTCACTATAGGttatcagccttatcatgtggGTTTTAGAGCTATGCTGAAA	61	sgRNA targeting *RDR6*

RDR6_2	GCGGCCTCTAATACGACTCACTATAGGtgaaggggttgatttcctggGTTTTAGAGCTATGCTGAAA	61	sgRNA targeting *RDR6*
RDR6_3	GCGGCCTCTAATACGACTCACTATAGGtgcaagaggaacgtgtgaggGTTTTAGAGCTATGCTGAAA	61	sgRNA targeting *RDR6*
sgRNA_R	AAAAAAAGCACCGACTCG	61	Universal reverse primer for sgRNA template amplification
**Template amplification**			
ADH1_TAF	CAGCTGCTATATAAATCCCCTTC	49	Amplification of the ADH1 cleavage template
ADH1_TAR	AGAAAACCAAAAGCGATGGA	49	Amplification of the ADH1 cleavage template
ADH1_TBF	CAGCTGCTATATAAATCCCCTTC	50	Amplification of the ADH1 cleavage template
ADH1_TBR	AAAGAGAATGGCGACTCGAA	50	Amplification of the ADH1 cleavage template
AGO4_TF	CCCTGCGTTGTTCTGAAAC	58	Amplification of the AGO4 cleavage template
AGO4_TR	GTGGCTTCTTTAATCTCTGGG	58	Amplification of the AGO4 cleavage template
CHLI1_TAF	TTAGCCACACAGCAATGGAG	52	Amplification of the CHLI1 cleavage template
CHLI1_TAR	TTCAGGCCAGGTAAACCAGT	52	Amplification of the CHLI1 cleavage template
CHLI1_TBF	GGCGAACTTCTCGGAAACTA	52	Amplification of the CHLI1 cleavage template
CHLI1_TBR	CGCATAAACGTCACCTCTAACA	52	Amplification of the CHLI1 cleavage template
NRPD1a_TF	CCTCAAAGCCGACTAGTTTC	58	Amplification of the NRPD1a-cleavage template
NRPD1a_TR	GCTTTGGTTTTTGTGTTCTGG	58	Amplification of the NRPD1a-cleavage template
NRPE1_TF	TGCAATGTGGGTTTTAAAGACG	58	Amplification of the NRPE1-cleavage template
NRPE1_TR	CATGATGCCACGAAAATACCA	58	Amplification of the NRPE1-cleavage template
PIE1_TAF	ATTTTCTACATCTGTTTGCGCC	49	Amplification of the PIE1 cleavage template A
PIE1_TAR	CAGCTACCGCATCAAGAGAA	49	Amplification of the PIE1 cleavage template A
PIE1_TBF	AGCAAGTGAGCTGAATCC	49	Amplification of the PIE1 cleavage template B
PIE1_TBR	ACCTTGGAAAACGGACTT	49	Amplification of the PIE1 cleavage template B
PIE1_TCF	AACAGCGTGTGAACTTGA	49	Amplification of the PIE1 cleavage template C
PIE1_TCR	GCACTAATTAATCTCACCTGG	49	Amplification of the PIE1 cleavage template C
PIE1_TDF	TGAGGCTGCAATGGAAAA	49	Amplification of the PIE1 cleavage template D
PIE1_TDR	CATCCACAATCATCAGCAT	49	Amplification of the PIE1 cleavage template D
PIE1_TEF	ATTTTCTACATCTGTTTGCGCC	50	Amplification of the PIE1 cleavage template E
PIE1_TER	TGCTTGTCCATAGCCTTCT	50	Amplification of the PIE1 cleavage template E
RDR2_TF	ACAGTACACCACAAAACACTCA	58	Amplification of the RDR2-cleavage template
RDR2_TR	GTAAGGTAAGCCCGAGAAAATG	58	Amplification of the RDR2-cleavage template
RDR6_TF	GAGTACCCTGTTGGAGAGC	58	Amplification of the RDR6 cleavage template
RDR6_TR	CCCTTTTTCGCATACTCACTA	58	Amplification of the RDR6 cleavage template

SUPPORTING INFORMATION LEGENDS:

Supplemental Figure 1: Schematic representation of the pDEECO entry vector

The region between the left and right borders of the T-DNA (LB and RB) consists of the spCas9 cassette (red, egg cell-specific promoter pEC1.2 / spCas9 ORF / Pea3A terminator), the seed marker cassette (green, oleosin promoter / oleosin ORF fused with the GFP ORF / OCS terminator), and the selection marker cassette (blue, conferring resistance to phosphinothricin). The vector contains a unique *Mlu*I restriction site that allows insertion of the multiplexed tRNA::sgRNA unit cassette shown above. Brown: U6-26 promoter; olive: tRNA; yellow: common scaffold gRNA sequence; blue: different target site-specific sequences; red: transcriptional terminator.

Supplemental Figure 2: *In vitro* assay to estimate cleavage efficiency of sgRNA/Cas9 complexes on additional PCR-amplified target DNA

Representative agarose gels after *in vitro* cleavage assays testing three different sgRNAs each targeting the Arabidopsis genes *RDR2*, *NRPE1*, *NRPD1a*, *RDR6*, *AGO4*, *ADH1*, and *CHLI1*. Templates incubated with recombinant Cas9 protein but lacking sgRNA (--) were not cleaved; addition of the sgRNA/Cas9 complex resulted in complete (+++), moderate (++), or poor (+) cleavage. Standard concentrations of Cas9, sgRNA, and cleavage template were 30 nM, 30 nM, and 3 nM, respectively, incubation time was 1 hr at 37°C. M = 1 kb marker.

Supplemental Figure 3: *In vitro* assay to estimate cleavage efficiency of sgRNA/Cas9 complexes in different reaction buffers

(A) Representative agarose gel after *in vitro* cleavage assays in different commercially available buffers, namely Orange buffer (O; Thermo Scientific), Tango buffer (T; Thermo Scientific), Fast Digest buffer (FD; Thermo Scientific c), CutSmart buffer (CS; NEB), T4 ligation buffer (T4; Thermo Scientific), T7 *in vitro* transcription buffer (T7; see Material and Methods) or H_2O. Three nM Cas9 protein were assembled without (--) or with 3 nM sgRNA PIE1_3 or PIE1_4 and incubated with 150 ng template for 1 h at 37°C, followed by inactivation for 15 min at 65°C. M = 1 kb marker. (B) Quantification of (A) for sgRNA PIE1_3 (grey) and PIE1_4

(black). Band intensities were calculated as loss relative to the intensity of the control band with the ImageJ GelAnalyzer tool.

Supplemental Figure 4: Test of *in vitro* incubation parameters for cleavage by the sgRNA/Cas9 complex

(A and B) ADH1 template was incubated at 37°C with Cas9-RNP reconstituted with either ADH1_1 (low efficiency) or ADH1_4 (high efficiency, see Supplemental Figure 2). (A) Different incubation time prior to inactivation. (B) Different concentrations of sgRNAs with standard concentrations of 30 nM Cas9 and 3 nM template, incubated 1 hr. (C) Performance of Cas9 with efficiently cleaving sgRNAs PIE1_3 and PIE_4 on PIE1 template and ADH1_4 on ADH1 template at different incubation temperatures. Omission of sgRNA and ADH1_4 sgRNA on PIE1 template are non-cleaving controls.

Supplemental Figure 5: Determination of pool size for amplification of mutated alleles after *in vitro* cleavage with Cas9/sgRNA complex.

(A) gDNA extracted from pools of 50 individual seedlings, in different ratios between mutant line atr2E3 and wild type, was digested overnight with Cas9-RNPs reconstituted with PIE1_3, PIE4, or ADH1_4 (control) sgRNA and amplified with the primers PIE_TEF and PIE_TER, prior to analysis by gel electrophoresis. (B) Overview of experimental workflow.

Supplemental Figure 6: Confirmation of the protocol for a different target gene

(A) *In vitro* assay to screen for mutated alleles of the *RDR2* gene. Following the same approach used to screen for *PIE1* mutants, *RDR2* sequence containing the target site of potential mutations was amplified from individual plants and incubated at two different temperatures with Cas9 reconstituted with sgRNA RDR2_3 (+) or with empty Cas9 (-). (B) Sanger sequencing confirmed the presence of deletions of different length in the plants pre-screened in (A), in homozygous or heterozygous genotypes.

Supplemental Table 1: Oligonucleotides and PCR annealing temperatures

Except the universal sgRNA reverse primer, each sgRNA primer consists of a T7 promoter sequence at the 5' end (upper case) for *in vitro* transcription, the specific sgRNA targeting sequence (lower case) and a 3' end scaffold sequence that binds to the Cas9 protein (upper case again). Primers in upper case only were used to amplify the cleavage templates containing the sgRNA target sites from genomic DNA.
